# Open-Source Automated Mapping Four-Point Probe

**DOI:** 10.3390/ma10020110

**Published:** 2017-01-26

**Authors:** Handy Chandra, Spencer W. Allen, Shane W. Oberloier, Nupur Bihari, Jephias Gwamuri, Joshua M. Pearce

**Affiliations:** 1 Department of Electrical and Computer Engineering, Michigan Technological University, Houghton, MI 49931, USA; handyc@mtu.edu (H.C.); swallen@mtu.edu (S.W.A.); swoberlo@mtu.edu (S.W.O.); 2 Department of Materials Science and Engineering, Michigan Technological University, Houghton, MI 49931, USA; nbihari@mtu.edu (N.B.); jgwamuri@mtu.edu (J.G.)

**Keywords:** 3-D platform, four-point probe, conductivity, indium tin oxide, ITO, open source hardware, libre hardware, sheet resistance, transparent conducting oxide, TCO, 72.20.-i 72.80.-r

## Abstract

Scientists have begun using self-replicating rapid prototyper (RepRap) 3-D printers to manufacture open source digital designs of scientific equipment. This approach is refined here to develop a novel instrument capable of performing automated large-area four-point probe measurements. The designs for conversion of a RepRap 3-D printer to a 2-D open source four-point probe (OS4PP) measurement device are detailed for the mechanical and electrical systems. Free and open source software and firmware are developed to operate the tool. The OS4PP was validated against a wide range of discrete resistors and indium tin oxide (ITO) samples of different thicknesses both pre- and post-annealing. The OS4PP was then compared to two commercial proprietary systems. Results of resistors from 10 to 1 MΩ show errors of less than 1% for the OS4PP. The 3-D mapping of sheet resistance of ITO samples successfully demonstrated the automated capability to measure non-uniformities in large-area samples. The results indicate that all measured values are within the same order of magnitude when compared to two proprietary measurement systems. In conclusion, the OS4PP system, which costs less than 70% of manual proprietary systems, is comparable electrically while offering automated 100 micron positional accuracy for measuring sheet resistance over larger areas.

## 1. Introduction

The spreading of the open source movement to science has achieved success as a research accelerator for many disciplines [1]. However, unlike the zero marginal cost of using free and open-source software (FOSS), open-source hardware development faces challenges arising from manufacturing costs [2]. Significantly, 3-D printing, using low-cost open-source self-replicating rapid prototypers (RepRaps), has helped overcome this challenge [3]. The RepRap project has enabled personal production using low-cost polymer-based materials, such as polylactic acid (PLA), acrylonitrile butadiene styrene (ABS) and high-density polyethylene (HDPE) [4,5]. Scientists and engineers in diverse fields have begun using RepRap 3-D printers to manufacture the open-source digital designs of scientific equipment [6,7,8] including: colorimeters and nephelometers [9], turbidimeters [10], phasor measurement units [11], optics and optical system components [12], liquid auto-samplers [13], medical equipment [14], microfluidic handlers [15], biotechnological and chemical labwares [16,17,18], mass spectroscopy equipment [19], automated sensing arrays [20], DNA nanotechnology lab tools [21], and compatible components for medical apparatuses such as MRI [22]. Digital sharing of designs brings researchers, educators and citizen scientists cutting-edge scientific tools at incredibly low costs [23] compared to commercial options. Open source scientific tools are freely available for governments, universities, corporations and laboratories to reproduce at usually between 90% to 99% less than the cost of conventional equipment [6]. Preliminary value analysis [24] shows that research funders (such as NSF and NIH) can see a return on such investments in the hundreds or thousands of percent [25]. To further drive down the cost of scientific research and harness the full capacity of open-source design, multiple research applications can be bundled together using an open-source 3-D printer itself as a scientific platform [26]. Past work has demonstrated a number of generic fluid handling and analytical techniques using this approach [26] and a 3-D microscope [27].

In this study, this approach is further refined to develop a novel instrument capable of performing automated large area four-point probe measurements of semiconductors for solar photovoltaic (PV) applications in both wafer and thin film form. First, the technical requirements of four-point probe systems are reviewed. Then the designs for the conversion of a RepRap 3-D printer to a 2-D four-point probe measurement device are detailed for both the mechanical and electrical systems. Free and open source software and firmware are developed to operate the tool and are described. To validate the capability of the circuit to measure a large range of sheet resistances, the device was tested on a wide range of discrete resistors. Then, indium tin oxide (ITO) samples of different thicknesses both pre and post-annealing were tested and the results were compared to a proprietary vendor’s four-point probe readings. Finally, 3-D mapping of sheet resistance of ITO samples were performed to demonstrate the device capability to measure non-uniformities in large-area samples. The results are discussed in the context of moving open source hardware development into complex characterization tool space for the semiconductor industry and future work is described.

## 2. Background

The PV industry has continued to grow and accelerate into most nations’ energy generation mix. In 2016, global solar installations have continued to grow and are projected to reach 64.7 GW [28,29]. Despite the growth, PV-generated electricity’s levelized costs need to be further decreased to out-compete the highly subsidized fossil fuel-based technologies and attain tera-watt deployment [30,31,32]. To further reduce levelized costs of solar electricity, more efficient PV devices need to be produced through the use of advanced light management schemes [33,34,35,36,37,38]. Future high-power-conversion-efficiency PV devices must effectively utilize the incident AM1.5 solar spectrum with negligible losses of incident photons into the cell. This requires new novel materials and techniques for the next-generation of solar cells [38,39]. However, more basic materials research especially on transparent conducting oxides (TCOs) as top contacts for PV devices is still needed. Ultra-thin TCOs, particularly indium tin oxide (ITO) still present some fabrication challenges [40]. There is on-going basic material research into advanced anti-reflection coatings [41,42] and transparent conducting oxides and electrodes [40,43,44]. Improved materials are required to augment performance, functionality, reliability and scalability of PV devices [45,46] and these materials are compared using resistivity as a core metric.

The most commonly used method to measure resistivity (or sheet resistance) of a thin-film of material is by using a four-point probe device [47,48,49,50,51,52,53,54,55,56,57]. The measurement functions by passing a current through the outer two probes, whilst simultaneously measuring the potential produced across the inner two probes. By calculating the ratio of voltage to current, the sheet resistance of the sample can be deduced [47,48,57]. The advantage of using the four-point probe method is that the method ignores contact resistance between the probe and material [47,53,57,58]. However, effects due to the geometry of the sample as well as the configuration of the probe often require correction factors to be applied to produce an accurate result [47,48,57,58,59,60,61]. The need to calculate correction factors can be avoided by employing the dual configuration method, which requires taking an extra measurement with the probes in a different configuration [54,56,62,63]. Reversing current applied to the current probes is also commonly employed to eliminate small offset voltages associated with thermoelectric effects [60].

Sheet resistance measurements are frequently used in PV research as a way to analyze the properties of PV materials during processing. It can be evident from a sheet resistance measurement alone if a substrate will produce a poor PV device and should be discarded. Imperfections during PV processing could result in non-uniform thickness. Therefore, it is recommended to perform measurements across the entire substrate of the sample [64]. Due to the number of measurement points required by this kind of testing, manual methods tend to be time-consuming. The steps in the characterization process such as: raising and lowering the probe head to different points on the sample, measuring forward and reverse currents, recording the result and zeroing the digital multimeter offset, often requires long time periods to perform. To improve the speed and precision of such testing, automated probe positioning devices can be employed. Automated testing systems available are expensive and proprietary. They are also often inadequate for emerging areas of study such as substrates with complex surfaces. Even if the required modification is minor, the closed-source nature of these device makes such modification prohibitively difficult or expensive [6]. In this study an open source system is described that overcomes these limitations.

## 3. Experimental Setup

### 3.1. Experimental Overview

This work demonstrates a low-cost open source automatic measurement system for sheet resistance. The developed system aims to perform automated measurement of sheet resistance of several points within a sample. Various existing open source hardware and software were utilized to make equipment development faster and more reliable. The system consists of a modified open source RepRap 3-D printer, a custom-designed measurement circuit, and graphical user interface (GUI) for a computer. A user enters data on the GUI about the geometry of the sample, points on the sample to be measured, current value, and center coordinate of the sample on the 3-D printer coordinate system. The precise center coordinate is not important as it is not possible to precisely position the sample (with 100 micron accuracy) on the print bed. The 100 micron accuracy discussed here is the distance between two points of measurement. Thus it should be pointed out that the uniformity of the conductivity values is in relation to the distance between two or more points on the sample, which are highly accurate rather than an absolute position on the sample. The GUI software then sends ASCII G-Code to the modified RepRap directly, as it has been pointed out previously that a RepRap 3-D printers can be used as 3-D motion control equipment for scientific experiments [26]. The RepRap, with a four-point probe head in place of a hot-end, is able to move the probe automatically to several points on the sample with positional accuracy of 100 microns. The GUI software controls the measurement circuit to perform the measurements automatically and saves the results to the computer as a CSV file. The inline four-point probe configuration is used for the system, with outer probes as the current probe and inner probes as the voltage probe. The system is intended to perform measurement with only one four-point probe configuration and will calculate approximate sheet resistance using equation:

R_sh_ = 4.532 V/I (1)

**Figure 1:**
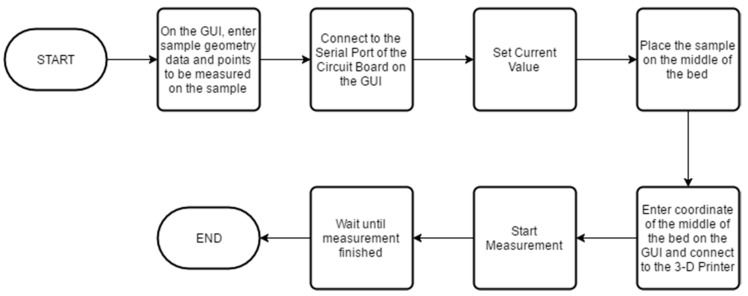
Measurement process flowchart.

V, I respectively are voltage measured on the inner probes and current through the outer probes. If the thickness of the sample is defined as t, distance between needles of the probe as s, diameter of the sample as d, and distance of the probe to the edge of the sample as *x*, then Equation (1) is valid only when s/t, d/s, and *x*/s are sufficiently large and the distance between each needle are equal [47,48,57,65]. Several similar systems have been developed [50,55,56], but none has tried to develop a system that will automatically measure different points on a sample, nor have the designs been disclosed in a digital format as done here for easy and inexpensive replication by other researchers. The measurement flowchart for the system is shown in Figure 1.

### 3.2. Equipment and 3-D Motion Control Description

**Figure 2:**
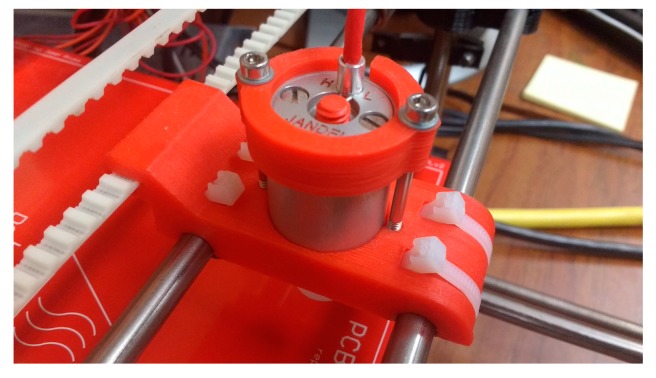
Printed four-point probe holder showing mounted four-point probe head.

**Figure 3:**
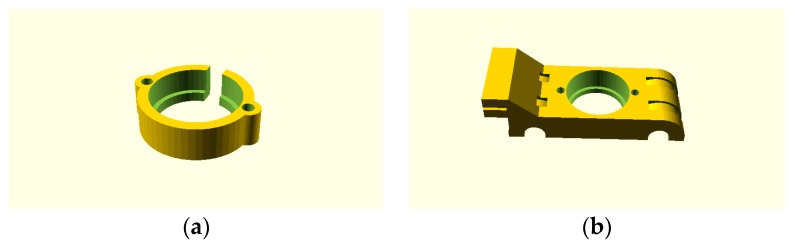
OpenSCAD images showing: (**a**) Probe holder top part; (**b**) Carriage part x-axis mount.

A Prusa Mendel (iteration 1), a RepRap open source 3-D printer, is used to provide precise positioning of the four-point probe on the sample in three-dimensional space. Such a printer has an x-y-z step resolution of 100 microns. A Jandel cylindrical four-point probe head [66], an inline four-point probe, is mounted on the 3-D printer in place of fused filament fabrication printer head. A four-point probe head customized for ITO films with 1 mm probe spacing, 500 µm probe tip radius, and made of tungsten carbide was used in this study [67,68]. A custom 3-D printed probe head holder was designed to attach the four-point probe head to the printer (Figure 2). The designed probe head holder has two parts. The first part is a four-point probe holder used to secure the four-point probe head to the carriage part using two screws (Figure 3a). The second part is a carriage part which slides around the x-axis smooth rods of the printer and is attached to the toothed belt (Figure 3b). The design of the carriage makes it possible for the printer to move the probe assembly around the x-axis smooth rods using linear bearings tied with cable ties underneath the holder.

**Figure 4:**
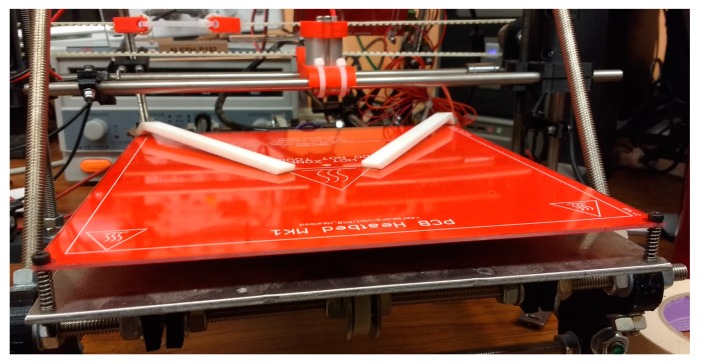
3-D printer bed fixed with springs and a pair of printed specimen clips (white).

The GUI is able to control the printer directly by sending ASCII G-Code over a USB connection, configured as an emulated serial port. Care must be taken to ensure that there is no z-wobble (change in x-y coordinate when z coordinate is changed) on the printer as this will possibly damage the sample when lowering the probe to the sample. A pair of specimen clips was also 3-D printed to hold the sample in place on the bed so that the sample will not move around and get scratched by the needles of the probe. The bed itself was fixed using springs so that precise z-positioning of the probe is not needed (Figure 4). This way the printer will lower the probe head all the way until minimum z-coordinate, and the force of the springs will ensure that the sample will touch the plastic pad on the probe. The sample must touch the plastic pad for proper operation of the probe as recommended by Jandel [66].

### 3.3. Open Source Measurement Circuit

The measurement circuit is designed with the aim to be a low-cost open source alternative to the more expensive commercialized equipment. As such, many design decisions are based on providing sufficient accuracy with low-cost hardware. The circuit consists of an adjustable current source, voltage measurement circuit, and an Arduino-compatible microcontroller board as the main controller for rapid development.

#### 3.3.1. Adjustable Current Source

**Figure 5:**
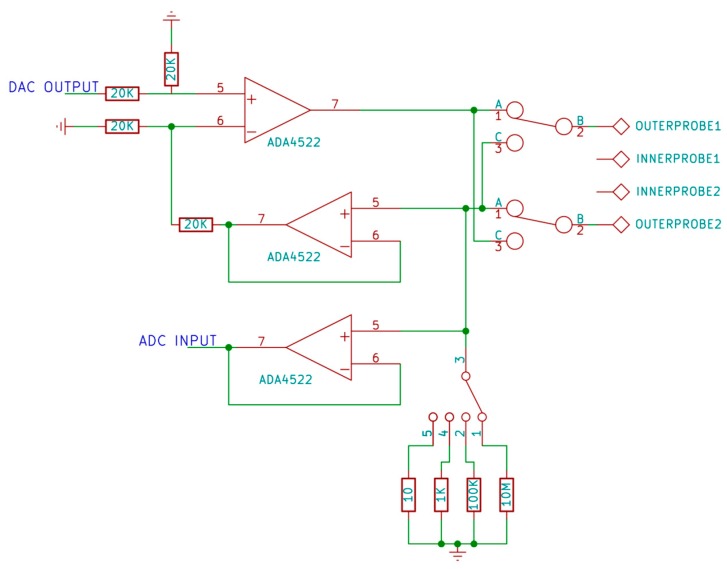
Current source circuit.

Sufficiently large current must flow through the sample so that the voltage generated between the two inner probes will be large enough to measure accurately [58]. Because contact resistance exists between the probe and sample [57,69], generating sufficiently large current means a large voltage drop will also exist on the contact resistance, potentially exceeding the current source voltage compliance. Because of this a current source with ~48 V voltage compliance is designed as seen in Figure 5. An operational amplifier ADA4522 from Analog Devices is chosen because of its 55 V operating voltage, required for designing a high voltage compliance current source, and because of its low bias, low offset, and zero drift, which are required for accurate sensing and amplification of small voltages [70,71].

Another design requirement is that the current source must be adjustable across a large range; from nanoampere to miliampere to accommodate a broad range of sheet resistance values. The combination of digital to analog converter (DAC) and a set of four resistors will allow a wide range of current value to be set. A difference amplifier circuit [72] will set the voltage across the set of four resistors to be the same as the analog voltage from DAC. Switching the active resistor together with changing the analog voltage value will allow a current range from 10 nA to 10 mA.

A set of four single-pole, single-throw (SPST) reed relays [73] are chosen to switch the set of four resistors because they feature a very low ON resistance and have virtually no leakage current, which minimizes error in the measurement. Another set of reed relays was also added to provide the capability to reverse current during measurements.

**Figure 6:**
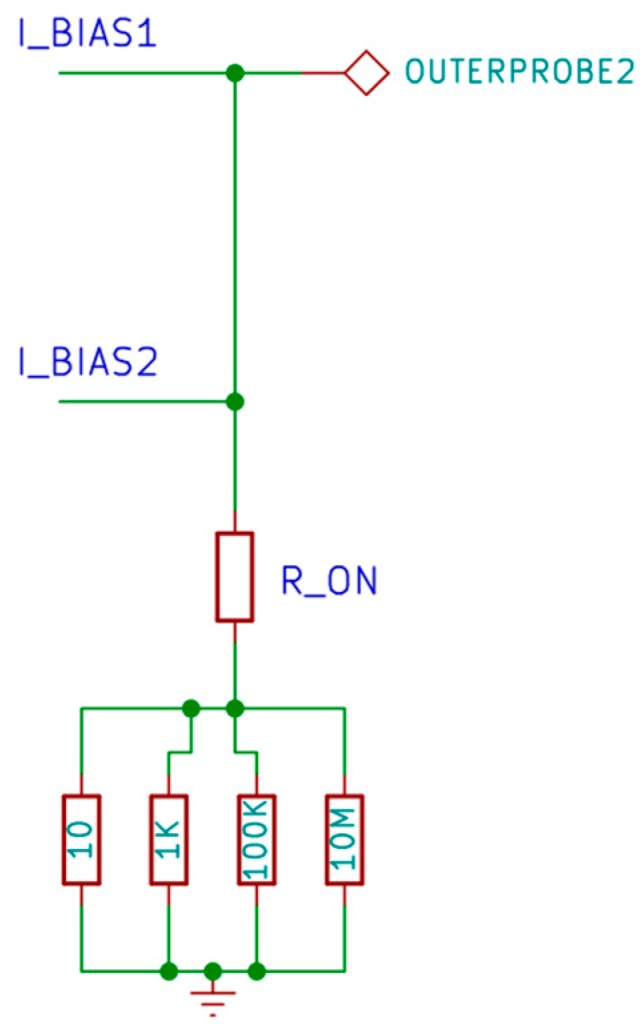
Parasitic resistance and leakage of current source circuit.

The actual value of current needs to be known for the calculation. Because of the limited capability of the current source circuit to set exact value of current, a feedback mechanism is introduced where the analog voltage on the set of resistors is read back by an analog to digital converter (ADC). The ratio between the analog voltage and the value of resistor will give the actual value of current. However, because of parasitic resistance due to ON resistance of the relay and current leakage due to input bias of op-amp (Figure 6), a slight error will occur when measuring the actual current. Current leakage may also occur because of insulation resistance of cables, probes, and the test fixture [74]. In general, the lower the current value needed to measure a sample, the greater the inaccuracy will be because a low current value is more susceptible to external noise. A simple RC low pass filter was added on the input of ADC to reduce signal noise when measuring the current value. Finally, the circuit is also capable of disabling the current source by setting the DAC voltage to zero. This is required when lowering the probe to prevent arcing between the probe and the sample, which could damage the sample and shorten the lifetime of the probe.

#### 3.3.2. Voltage Measurement Circuit

**Figure 7:**
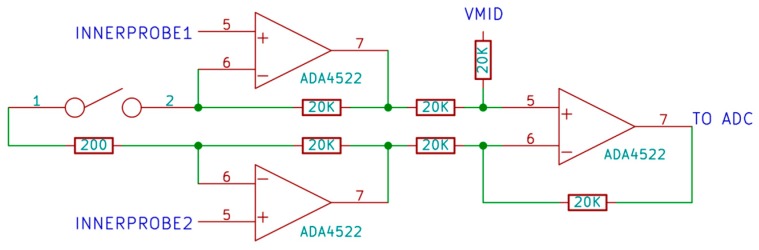
Instrumentation amplifier circuit.

The voltage measurement circuit should be able to measure small voltage differences accurately and should draw very little current. Thus, an instrumentation amplifier circuit [75] is designed with selectable gain between 1 and 200 by switching a set of two resistors using a reed relay (Figure 7). A differential ADC on the Arduino-compatible board is used to measure the output of instrumentation amplifier circuit with respect to a voltage V_mid_, which is half of the ADC reference voltage. This makes it possible to measure voltage on the inner probe for both forward and reverse current configuration.

Imprecise values of the gain resistor, finite ON resistance of reed relays and imprecise matching of resistors in the instrumentation amplifier circuit will contribute to gain error in the circuit. To minimize the error, a 0.01% tolerance resistor is used in the circuit. An RC low pass filter was also added on the output of this circuit to reduce noise on the measurement results.

#### 3.3.3. Main Controller

**Figure 8:**
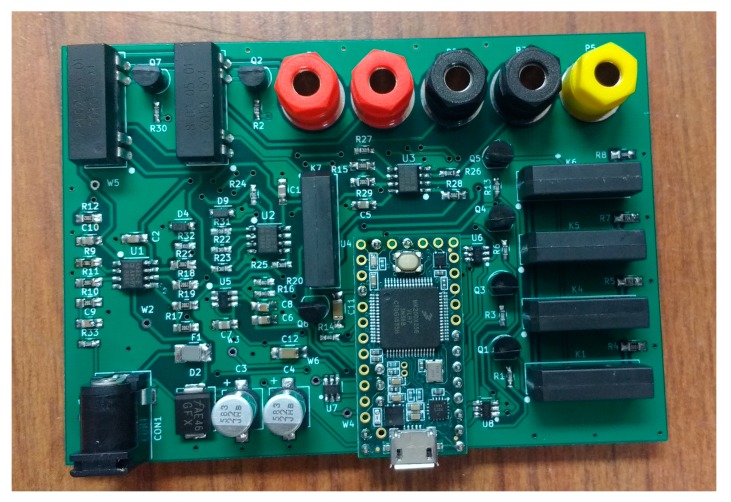
Assembled open source printed circuit board for four-point probe measurements.

Teensy 3.2, an Arduino-compatible board powered by a Freescale MK20DX256VLH7 chip (32-bit ARM microcontroller), was chosen as the main controller for the circuit. The need to develop low-cost open source equipment and time constraints during the development influenced the decision to use this board. The chip provides on-board 12-bit DAC, 16-bit single-ended and differential ADC which is needed for the current source and voltage measurement circuit, respectively. Arduino is also suitable for rapid prototyping because of readily available libraries and boards [6,76]. Initially, the circuit was prototyped on a breadboard to verify the circuit operation. The printed circuit board version of the circuit was designed using KiCad EDA [77]. Next, the designs were fabricated and assembled into a fully assembled circuit (Figure 8). All of the KiCad schematics and printed circuit board files needed to make both a breadboard circuit and printed circuit board version are available at the Open Science Framework [78].

### 3.4. Firmware

A custom firmware was written using the Arduino Integrated Development Environment (IDE) and open source libraries for rapid development. The firmware was designed to receive and reply to commands over a USB connection via GUI software running on a PC. Several features of the firmware include: detecting whether samples are connected to the probes, disabling and enabling the current source, automatically detecting suitable current levels for the connected sample, automatic gain setting for instrumentation amplifier, switching forward or reverse current configuration, and also include a digital low pass filter to reduce noise from the measurement results. The auto current feature will select among these current values: 10 nA, 100 nA, 1 µA, 10 µA, 100 µA, 1 mA, 10 mA, that will generate sufficient voltage between the two inner needles when the sample is touching the probe. The firmware is released under a GNU FDL and can be downloaded from [79]. An attempt to reduce the effects of ON-resistance of the reed relay is done by adding the value of measured ON-resistance into the firmware calculation.

### 3.5. Software and Graphical User Interface (GUI)

**Figure 9:**
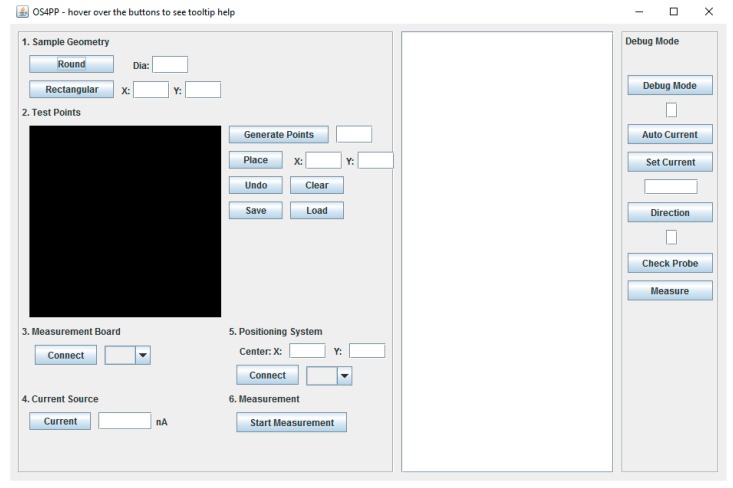
The graphical user interface (GUI) for open source four-point probe (OS4PP) software.

The Java swing toolkit, included in the Java Development Kit [80], is used to develop the GUI because they are platform independent and the GUI could be rapidly generated using tools such as WindowBuilder [81]. The GUI could be designed easily using drag and drop methods, and uses a “what you see is what you get” (WYSIWYG) interface of this open source tool. The finished user interface is shown in Figure 9.

The user interface is divided into three parts: the measurement settings in the left part, text display in the middle part, and an area to debug commands on the right. The measurement settings are straightforward to use, with numbered steps to follow. Users can enter which point on the sample to measure manually or it could be generated automatically. The measurement process and result can be seen in the text display. The software will automatically turn off the current source before lowering the probe head, and then turn it on again after touching the sample. This is done to prevent arcing between the needles and the sample. The software will control the printer to move the probe head through all programmed points. The coordinate of each point, current and voltage measured is stored as a CSV file as the measurement process is ongoing and can be exported after the characterization process is complete. The GUI is released under GNU FDL and can be downloaded from [78,79].

## 4. Validation

**Figure 10:**
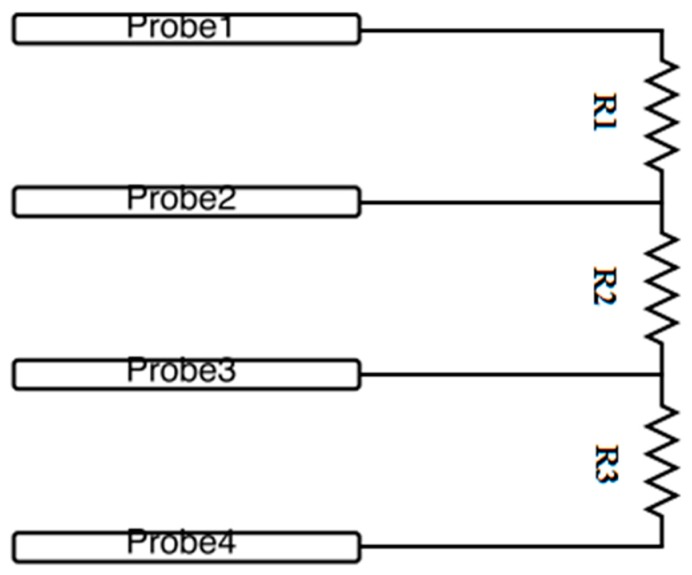
Discrete resistor measurement circuit.

The developed system is designed to be able to measure sheet resistances of various materials, but depending on the probe and material itself, a large contact resistance could occur. The current source would be unable to supply enough current to generate sufficient voltage between the two inner probes due to a limited compliance voltage. Therefore, to validate the capability of the circuit to measure a wide range of resistances, contact resistance can be avoided by measuring different values of discrete resistors. The test configuration can be seen in Figure 10. It should be noted that the resistors do not need to be equivalent in Figure 10. The method is only measuring the central resistor, R2.

The value of the resistors for validation are 10 MΩ, 1 MΩ, 100 KΩ, 10 KΩ, 1 KΩ, 100 Ω, and 10 Ω. Current source values are set so that sufficient voltage is generated on the resistor. The measured value using the open source four-point probe circuit is compared with the measured value by a Fluke multimeter model 187 whose accuracy specification is up to 1% + 4 for resistances up to 32 MΩ. The comparison verifies that the circuit is able to measure a wide range of sheet resistances, namely from mega ohm range to tens of ohm.

The intended purpose of the developed system is to measure ITO samples. To this effect, the second validation was performed on a set of ITO samples, which are: Jandel ITO reference sample 12.58 Ω/square and three 50 nm thick ITO samples on glass annealed for 10, 20, and 30 min respectively using UHP forming gas (FG) (95% N_2_/5% H_2_ from air gas) in a sealed (by vacuum coupling components) quartz tube inside a tube furnace. The annealed samples were deposited on glass substrates using RF sputter deposition techniques as described in [40]. The measurement results from the open source four-point probe are then compared to results obtained from two other commercial four-point probe systems (Jandel RM3 Test Unit with Lucas Lab probe station, and a system comprising of separate Keithley Current Source Model 220 and Keithley Multimeter Model 196). The measurement method for the three films were performed by measuring 30 random points throughout the samples. Standard deviation from each sample is also calculated to show the range of thickness variation of the film sample.

The last validation is to perform automated measurement on multiple points on the reference sample and also on the three annealed 50 nm samples. There were 100 points automatically generated on the GUI and distributed uniformly over an area of 40 × 40 mm^2^ located at about the center of the reference sample. To avoid correction factor calculations, the points were made to be at least 10 probe spacings away from the edge of the sample, with the probe spacing being 1 mm. Because of the small size of the three annealed samples, only 40 points were measured over an area of 10 × 10 mm^2^. The measured sheet resistance values are then used to map the sample surface using surface plots to show the degree of variation in film uniformities over the sample surface.

## 5. Results and Discussion

**Figure 11:**
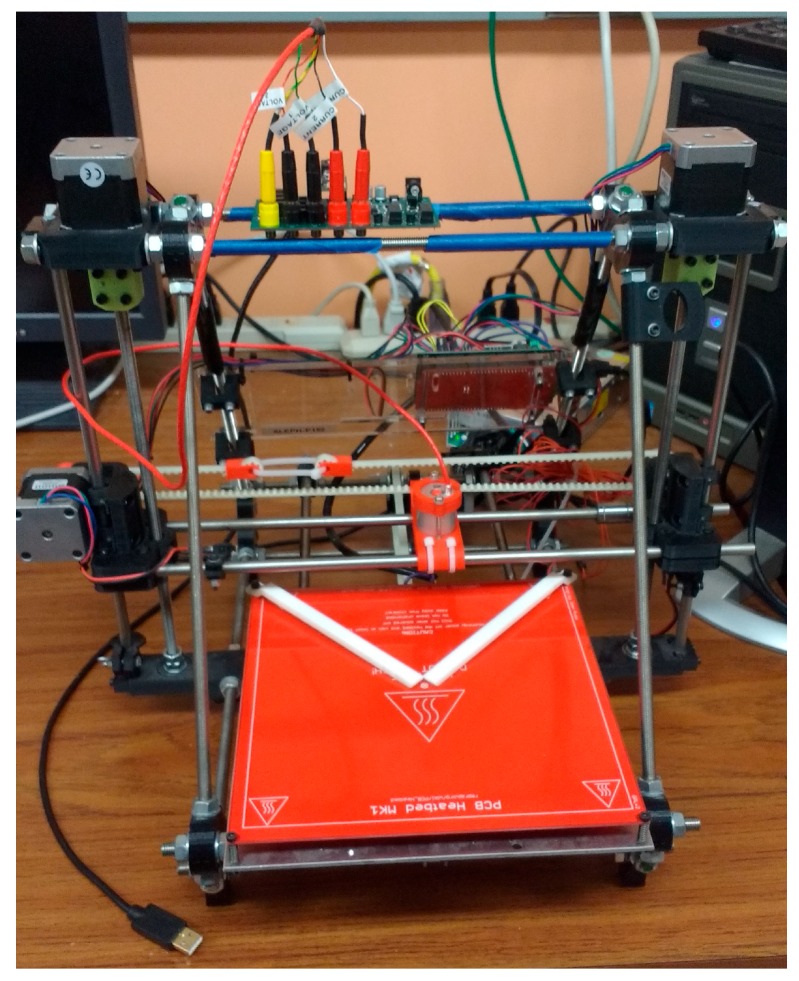
Modified 3-D printer with custom measurement circuit for OS4PP measurements.

The full OS4PP system consisting of modified printer connected to the measurement circuit can be seen in Figure 11.

**Figure 12:**
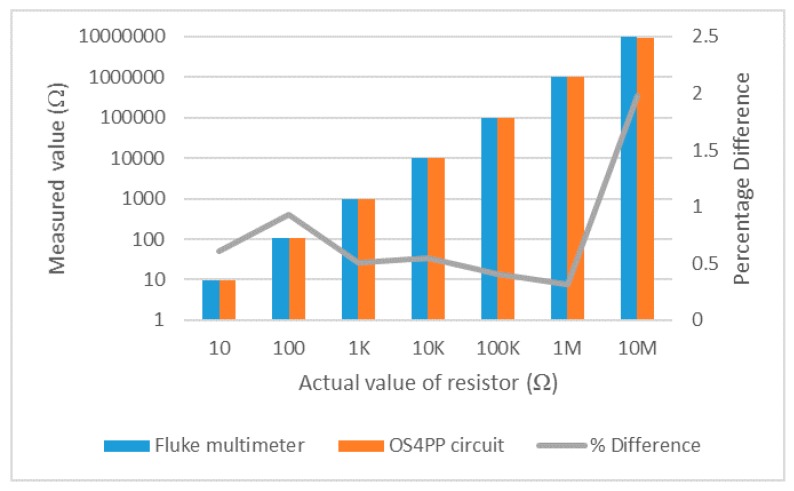
Comparison of measurement results on discrete resistor.

The results of the resistor measurement using the OS4PP system and fluke multimeter can be seen in Figure 12. The comparison of measurement results of resistors from 10 to 1 MΩ show errors of less than 1%, while measurement result of 10 MΩ resistor shows an error of about 2%. Measuring 10 MΩ resistor requires a very low value of current, on the order of nanoampere, which makes it more susceptible to noise. Furthermore, high-resistance materials do not allow the charge to decay quickly, resulting in unstable measurements.

Table 1 shows measurements of ITO samples using the three different equipment types. The results measured from three different equipment/systems show varying amounts of standard deviation due to non-uniform variations in ITO film thickness and different measurement points for each sample. The standard deviation for sheet resistance measurements for the reference samples using different systems is observed to be consistently lower, and the sheet resistance values can be considered to be constant and within the margin of error. This cannot be used as a comparison for accuracy, because the samples are non-uniform and the choice for each points for different equipment also vary. The results presented in Table 1 show the effect of annealing time on the resistivity of ultra-thin ITO films and are in agreement with results presented in refs [40,45]. There is an observed general trend of decreasing R_sh_ with increasing annealing time and the lowest R_sh_ value is attained for films annealed for 20 min beyond which the R_sh_ is observed to increase. Despite showing a high standard deviation value for the 50 nm film annealed for 30 min, the results indicate that all measured values are within the same order of magnitude when the three types of equipment are compared. In summary, the OS4PP system (with ITO optimized tips) is comparable with the commercial four-point probe systems.

**Figure 13:**
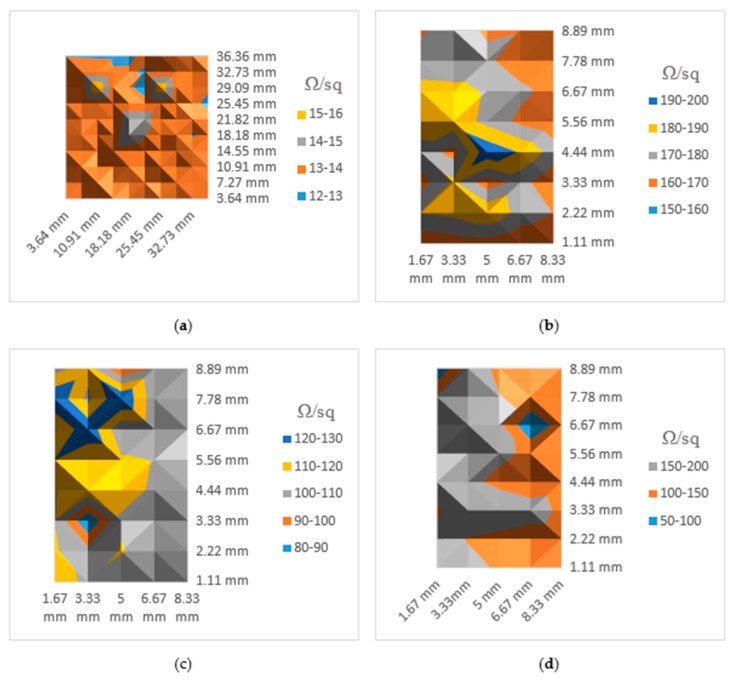
Results of automated sheet resistance measurement (Ω/sq) using OS4PP: (**a**) Reference sample; (**b**) 50 nm ITO annealed 10 min; (**c**) 50 nm ITO annealed 20 min; (**d**) 50 nm ITO annealed 30 min.

Figure 13 shows automated measurement results using the OS4PP. A point in coordinate (10.91, 29.09) on the ITO reference sample had a sheet resistance value 27.2 Ω and had to be rescaled. There were visible surface defects on these samples because of repeated experiments which explains why a point on the reference sample had very high sheet resistances compared to the rest of the points. Also there is a possibility that annealing the film for 30 min might have triggered some transformation in the morphology of the film resulting in area on the sample surface having isolated islands due to agglomeration. The high value of standard deviation is also seen while using the commercial systems, further proving that inconsistencies in the thin film may have caused the OS4PP to record real values.

The automated measurement results also confirm results in Table 1, where standard deviation in Table 1 is proportional to the uniformities of the sample. Samples in Figure 13a,c do not vary as much as the other two, which is why their standard deviation is lower. The measurements are also very sensitive to dust, dirt, or other foreign substances on the sample. Generally, the same measurements are taken multiple times to make sure the results are not affected by bad contact to the sample. Normally such tests would be done in a clean room directly after deposition, thus removing this source of error. However, the lack of an in situ sheet resistance measurement system post-deposition gives valuable information. Further, this tests for film integrity over long periods of time. It is known that oxygen absorption in ITO films affects conductivity and transparency [82,83]. Hence, there is a need for an automated system that can measure this change in sheet resistance ex situ without the need for repeated intervention by a skilled technician. Further, the automated nature of the OS4PP ensures that the same point is measured each time for longitudinal studies. This mitigates the disadvantage of commercial equipment where the probe is manually placed and is subject to operator error.

Taking the results of three validations into account, the developed open source system performs well in comparison to other commercial systems and could be used as a substitute to conventional equipment in applications requiring automated measurements of large number of points on the samples. The resistor validation shows how accurate the system could be, less than 1% error for sheet resistances smaller than 1 MΩ. The OS4PP performs better at measuring lower values of sheet resistances because higher value of current could be used for these samples, increasing the signal to noise ratio. Actual sample measurements are complicated by variability in contact quality between the probe and samples, and as such taking multiple measurements and averaging them is recommended. The developed OS4PP is compared to Jandel four-point probe system in Table 2 to show the differences in features, capabilities, and costs. Calculating the total cost of probe head, circuit, and 3-D printer shows a 73% reduction in cost from the manual commercial four-point probe. It should be pointed out here that the area the OS4PP can cover is much larger than the Jandel as can be seen in Table 2. In addition, and perhaps most importantly, the OS4PP is automated with x-y positional accuracy of 100 microns while the more expensive commercial system is not automated and involves manual placement of the sample. The open source nature of this project also allows researchers to study how the tool works and modify it according to their specific research needs. As this project consists of several open source hardware and software projects, any part could be modified, replaced or repaired if damaged.

There are several ways the OS4PP can be improved. The accuracy of the tool depends on how accurate the set of four shunt resistors are and how small the effects are of the ON-resistance of the reed relay. Actual sample measurements could vary depending on the contact quality from the probe to the samples and also because of dust and foreign substances on the samples. In general, good ITO films have lower sheet resistance values (a few kΩ/□ or less), hence such films can be characterized with much greater accuracy. There is, however, the need to continue improving the performance of the designed custom measurement units. Proper steps must be taken to minimize the effects of electrostatic fields, leakage currents and temperature among others. Electrostatic shields can be built to enclose the sensitive circuitry and the cabling in the circuit. Substituting the unshielded reed relay into a shielded one could also help reduce the noise in the measurement. Using good quality insulators, reducing humidity, and using guarding may minimize the effects of leakage current [74]. Minority/majority carrier injection is also a problem that could be minimized by keeping the voltage generated between the two inner needles low [74]. Finally, a more accurate ADC will be needed to sense low voltages accurately. Additionally, a calibration method might be required to eliminate the effects from finite ON resistance of relays.

## 6. Conclusions

Research in basic electronic materials for solar photovoltaic cells and other applications is hampered by the costs associated with electrical measurement of the materials. In this paper, an open source methodology has been applied to electrical conductivity measurements to solve challenges in basic materials research by reducing the cost required for scientific characterization equipment. Digital sharing of the designed scientific equipment combined with open source 3-D printers have made such open source scientific equipment easier and faster to develop. The open source nature has also made it easier to customize the equipment for different applications or fixing the equipment when there are problems. In addition, the open source documentation is all readily available so researchers will know exactly how it works, and there is no ambiguity as to how measurements are made.

The use of the self-replicating rapid prototyper (RepRap) printer, together with a custom measurement circuit, have been investigated to provide a low-cost, open source alternative to expensive automated sheet resistance measurement equipment. A four-point probe head has been installed in place of a 3-D printer head and controlled with software. A custom-designed measurement circuit together with graphical user interface (GUI) software were developed to measure sheet resistance of ultra-thin indium tin oxide (ITO) film samples. The validation shows that the circuit could measure sheet resistances smaller than 1 MΩ with less than 1% error. The results indicate that all measured values are within the same order of magnitude when compared to two proprietary measurement systems. This compares favorably with proprietary commercial systems. In addition, the open source four-point probe (OS4PP) developed here automates the measurement of sheet resistivity. The results of 3-D mapping of sheet resistance of the ITO samples successfully demonstrated the automated capability to measure non-uniformities in large-area samples. This is generally done manually when using manual four-point probe stands, which has far less positional accuracy than the 100 micron resolution shown here. The automation process can help in film surface mapping in a short period of time thereby promoting the efficient utilization of resources. In conclusion, the OS4PP system, which costs less than 70% of manual proprietary systems, is comparable electrically while offering automated 100 micron positional accuracy for measuring sheet resistance over larger areas.

## Figures and Tables

**Table 1:** Measurement results of several indium tin oxide (ITO) samples on different equipment.

Sample	OS4PP		Jandel		Keithley	
	R_sh_ (Ω/sq)	Std Dev	R_sh_ (Ω/sq)	Std Dev	R_sh_ (Ω/sq)	Std Dev
R. Reference Sample	13.308	0.49	13.09	0.85	13.304	0.27
A. 50 nm ITO annealed 10 min	171.86	11.79	182.36	15.21	176.08	8.4
B. 50 nm ITO annealed 20 min	110.76	7.33	113.42	9.18	111.8	5.83
C. 50 nm ITO annealed 30 min	142.84	27.76	177.52	42.79	159.07	15.9

**Table 2:** Comparison of commercial proprietary machine with OS4PP.

Component	Commercial Machine	Open-Source OS4PP
Probe	Jandel macor probe head	Jandel cylindrical four-point probe head
Measurement Unit	RM3 test unit	Custom measurement circuit
Mounting Stand	Lucas labs S–302−4	RepRap Prusa Mendel i1 with custom probe holder
Source	Closed source	Reprap project + osf.io
Cost	$1600 probe head + $3200 RM3 + $2500 S–302−4 = $7300	$1600 probe head + $130 circuit + $250 RepRap = $1980
Maximum Wafer Size	4” circular	8” × 8” square
Accuracy	0.3% (high sensitivity mode)	1% (<1 MΩ sheet resistances)
Positioning Resolution	Manual sample placement by hand	100 microns X-Y accuracy
Multiple Measurement	Manual	Automatic
